# Combination Immunotherapy: Taking Cancer Vaccines to the Next Level

**DOI:** 10.3389/fimmu.2018.00610

**Published:** 2018-03-22

**Authors:** Jeremy M. Grenier, Stephen T. Yeung, Kamal M. Khanna

**Affiliations:** ^1^Department of Immunology, University of Connecticut Health, Farmington, CT, United States; ^2^Department of Microbiology, New York University Langone School of Medicine, New York, NY, United States; ^3^Perlmutter Cancer Center, New York University Langone Health, New York, NY, United States

**Keywords:** cancer vaccines, program cell death, CD8-positive T-lymphocytes, tumor, immunotherapy

## Abstract

With the advent of checkpoint blockade therapies, immunotherapy is now a critical modality for the treatment of some cancers. While some patients respond well to checkpoint blockade, many do not, necessitating the need for other forms of therapy. Vaccination against malignancy has been a long sought goal of science. For cancers holding a microbial etiology, vaccination has been highly effective in reducing the incidence of disease. However, vaccination against established malignancy has been largely disappointing. In this review, we discuss efforts to develop diverse vaccine modalities in the treatment of cancer with a particular focus on melanoma. Recent work has suggested that vaccines targeting patient-specific tumor mutations may be more relevant than those targeting unmutated proteins. Nonetheless, tumor cells utilize many strategies to evade host immunity. It is likely that the full potential of cancer vaccination will only be realized when vaccines are combined with other therapies targeting tumor immunoevasive mechanisms. By modulating inhibitory molecules, regulatory immune cells, and the metabolic resources and demands of T cells, scientists and clinicians can ensure vaccine-stimulated T cells are fully functional within the immunosuppressive tumor microevironment.

## Introduction

With the recent clinical successes of T cell checkpoint inhibitors, immunotherapy has become an effective, standard therapy for several cancers. These triumphs have reinvigorated the entire field of cancer immunotherapy and brought it into the limelight.

For decades, clinicians and scientists have attempted to develop methods to stimulate the immune system to target malignant cells. While many studies have shown that patients can develop immune responses against tumor antigen, the vast majority have yielded little clinical benefit (1–3). The reasons for this are multifactorial, and several possible explanations for the inefficiency of cancer vaccines have come to light in recent years. Choice of target antigen is a major determinant in the immunogenicity of a vaccine. However, increasing evidence has also shown that the tumor microenvironment has several mechanisms to interfere with immune cell function. Current data suggest that, like chemotherapy, effective cancer vaccination protocols will require combination immunotherapies to overcome tumor immunoevasion. In this review, we will discuss different vaccine formulations targeting two classes of antigens with a particular focus on melanoma. In addition, we highlight mechanisms by which tumor cells evade T cell immunity. Emerging data suggest that combining tumor vaccination with other therapies targeting immunosuppressive pathways may fully unleash the potential of cancer vaccines.

## Vaccines Targeting Shared Antigens

Perhaps the most important decision in designing a cancer vaccine is the choice of target antigen. Excluding non-targeted vaccines such as tumor lysate vaccines, the vast majority of tested vaccines have been designed to generate T cell responses against shared tumor antigens, that is, antigens expressed both by malignant cells and by non-vital healthy tissue (2). These shared antigens are generally overexpressed in malignant cells, but expressed at lower levels in healthy tissue or in early embryogenesis. Examples include melanoma differentiation antigens, antigens normally expressed during embryogenesis, and testes-associated antigens. Because these antigens are non-mutated self-proteins, high avidity T cells recognizing these antigens are likely deleted during development because of antigen recognition within the thymus leading to deletion of self-reactive T cells (4, 5). Thus, any vaccine platform targeting these antigens begins at a disadvantage by the limited repertoire of naïve T cells available to respond to the vaccine.

Nonetheless, clinical studies have shown that it is possible to stimulate T cell responses against shared antigens (1, 2, 6). An early study in melanoma patients showed that vaccination with the shared antigen gp100 induced weak gp100-reactive T cell responses, but altering anchor residues within the peptide significantly increased its immunogenicity by increasing MHC-I binding affinity (7). In a clinical study utilizing this strategy, a modified gp100 peptide vaccine given with IL-2 generated a detectable gp100-reactive T cell response in peripheral blood of melanoma patients (1). Furthermore, in a phase III study testing the same approach, patients receiving the vaccine with IL-2 had a higher overall clinical response rate and slightly longer progression-free survival compared to IL-2 alone (2.2 vs. 1.6 months) (8).

Peptide vaccines are not the only vaccine modality to be tested in the treatment of cancer. Dendritic cells (DC) have been used as vaccine platforms to stimulate antitumor T cell responses (9). An early study by Banchereau et al. showed that CD34^+^ progenitor-derived DCs loaded with peptides from MelanA/MART-1, MAGE-3, gp100, and tyrosinase could generate detectable T cell responses to some of these antigens (10, 11). Following this work, Palucka et al. showed that monocyte-derived DCs pulsed with allogeneic tumor lysate could generate T cell responses to shared tumor antigens, leading to a complete response in one patient and a partial response in a second (out of 20 vaccinated patients) (12). Larger clinical studies are underway to determine the clinical efficacy of this vaccine strategy in many other cancers (13).

## Vaccines Targeting Neoantigens

More recent work has focused on another class of tumor-associated antigens termed neoantigens. This class of antigen consists of “non-self” peptides that arise from non-synonymous mutations within the tumor genome. These mutations are generally unique to each individual patient tumor and, thus, represent an extreme in personalized medicine (14); however, the discovery of the so called “shared” neoantigens (i.e., mutated epitopes seen in more than one individual patient) is an exciting prospect, making neoantigen vaccines potentially more feasible (15, 16). Several studies have suggested that neoantigens may be responsible for the clinical efficacy seen with checkpoint blockade and some forms of adoptive cell therapy (17–22). Because neoantigens are uniquely expressed by tumor cells, high avidity T cells recognizing these epitopes will likely have escaped deletion in the thymus. Hence, vaccines targeting these antigens bypass a major obstacle obstructing vaccines against shared antigens. At the same time, vaccines against neoantigens should reduce off-tumor, on-target autoimmunity because of the tumor-specific expression of antigen.

Important work in mouse models has demonstrated the power of targeting immunotherapies to neoantigens (23–26). Castle et al. showed in a highly immunosuppressive mouse melanoma model that peptide vaccination against mutated sequences within Actn4 and Kif18b could delay B16 tumor growth (25). However, vaccination alone was insufficient to cure the mice (25). Similarly, Yadav et al. showed that peptide vaccination against mutated Dpagt1, Reps1, and Adpgk delayed tumor growth in the MC38 murine colon cancer model (23). In this study, neoantigen-specific T cells expressed high levels of the inhibitory receptors PD-1 and Tim3 within the tumor, suggesting T cell dysfunction. Peptide vaccination reduced the expression of these receptors within the tumor but this may be a result of the adjuvant used in the study (23). Using a different algorithm to predict immunogenic neoantigens, Duan et al. showed that vaccination against a neoepitope within Tnpo3 induced tumor regression in a prophylactic setting, and combination therapy with anti-CD25 or anti-CTLA4 improved the response to neoantigen vaccination (26). These preclinical studies highlight the burgeoning effort to reliably predict immunogenic neoantigens within individual tumors.

Several attempts have already been made to test neoantigen-specific vaccines in patients. Carreno et al. vaccinated three melanoma patients with neoantigen peptide-loaded DCs and showed that vaccination increased the magnitude and breadth of neoantigen-specific T cells (27). More recently, two groups have tested this approach using peptide and RNA vaccines. Ott et al. vaccinated six high-risk melanoma patients with 13–20 long peptides containing tumor-specific mutations predicted to bind HLA-A or HLA-B (28). Surprisingly, the majority of vaccine-stimulated T cells were CD4^+^ rather than the predicted CD8^+^ population. Nonetheless, four of the six patients showed no tumor recurrence 25 months later. Two patients with remaining disease were treated with pembrolizumab and subsequently had complete responses (28). Sahin et al. vaccinated patients with stage III and stage IV melanoma with synthetic RNAs containing up to 10 predicted neoantigens (29). In contrast to the study by Ott et al., Sahin et al. chose mutated sequences predicted to bind to both HLA class II molecules as well as HLA class I (29). Like Ott et al., this study also showed a preferential activation of CD4^+^ T cells in response to vaccination (29). Clinically, two patients had objective responses following vaccination. One patient displayed progression of disease following vaccination but had a complete clinical response following treatment with pembrolizumab (29). This work is highly encouraging, suggesting that tumor-specific vaccines are indeed immunogenic in patients. Combining vaccination with other forms of immunotherapy may greatly enhance the efficacy of treatment. Several clinical trials are also currently underway assessing the safety and feasibility of neoantigen vaccines in combination with radiotherapy or chemotherapy in patients with glioblastoma or breast cancer (NCT02287428; NCT02510950; NCT02427581; NCT02348320). As immunogenicity prediction algorithms become more accurate, vaccinating against this class of antigens may completely replace efforts to target shared antigens for the advantages listed above.

While determination of ideal tumor antigens is a high priority for researchers, the optimal vaccine formulation is still unknown. Many groups have explored recombinant nucleotide, peptide, or microbial vector-based vaccine strategies with varying success, but differences in targeted antigen and tumor type make direct comparison of different vaccine platforms difficult (2, 30). To address this gap in knowledge, the international Human Vaccines Project aims to determine the most immunogenic vaccine platforms for use in cancer patients by comparing different delivery methods targeting the same antigen (31). Data from such studies could greatly enhance the therapeutic benefit of future cancer vaccines. At the same time, the Human Vaccines Project has set another goal of better characterizing the immune fitness of patients with cancer (31). An early study by Almand et al. showed that DCs from patients with advanced cancer are poor T cells stimulators compared to cells from healthy controls (32). A more recent study also showed that peripheral blood from melanoma patients contained increased frequencies of myeloid-derived suppressor cells (MDSCs) and an immunosuppressive BDCA1^+^CD14^+^ DC subset which inhibits T cell activation in an antigen-dependent mechanism (33). These studies highlight the global immune dysfunction seen in patients with cancer. Further characterization of this immune dysfunction may help improve vaccine responses in this patient population.

## Combining Vaccines with Checkpoint Blockade

Many vaccine platforms are effective in generating detectable T cell responses against shared antigens or neoantigens. However, these T cells are often incapable of ablating established tumors. Many studies have revealed that tumor-infiltrating T cells are less functional than those found in circulation, both in vaccinated and unvaccinated patients (34–37). It is becoming increasingly clear that tumors utilize many strategies to evade antitumor T cells (38). Thus, tumor cell killing may be blunted by checkpoint receptor signaling, highly suppressive regulatory cells, or alterations in tissue oxygenation and nutrient availability.

Activated T cells express inhibitory receptors on their surface, limiting the magnitude of the T cell response and collateral tissue damage during normal immune responses. There are currently two T cell checkpoint pathways targeted by FDA-approved therapies: CTLA-4 and PD-1. CTLA-4 is a member of the immunoglobulin superfamily and limits T cell function when it binds CD28 on DCs during T cell priming (39). Ipilimumab, an antibody targeting CTLA-4, is an effective therapy for metastatic melanoma, receiving FDA approval in 2011 (40, 41). Nivolumab and atezolizumab are antibodies targeting PD-1 and PD-L1, respectively (42). When it binds the cognate ligand PD-L1, PD-1 limits antitumor T cell costimulation by disrupting CD28 signaling (43). The power of this inhibitory pathway in cancer is illustrated by the remarkable clinical responses seen in some patients treated with nivolumab (44, 45).

Importantly, PD-L1 expression is regulated by CD8^+^ and Th1 CD4^+^ T cell-produced IFNγ (46–48). This upregulation of PD-L1 in response to T cell attack is a highly conserved pathway and can explain much of the PD-L1 expression seen in certain cancers (49). As a result of this pathway, vaccine-induced T cells can also enhance the expression of PD-L1, and this can limit vaccine efficacy (50, 51). However, vaccine-induced PD-L1 expression does not reliably predict responses to PD-1 blockade in all tumor models (52). Two recent preclinical studies showed that PD-L1 expression on DCs and macrophages is more predictive of response to PD-1 blockade than PD-L1 expression on tumor cells themselves (53, 54). PD-L1 expression on DCs is known to limit T cell activation (55). Thus, PD-1 blockade may also influence antitumor T cell priming. Whether PD-1 disrupts priming of naïve antitumor T cells or produces an exhausted state in previously activated cells is a point of future inquiry (56).

Regardless of the exact mechanism, tumor vaccination and checkpoint inhibition may be a potent combination in cancer therapy. In an early preclinical study, CTLA-4 blockade delayed B16 tumor growth when combined with a GM-CSF-secreting tumor vaccine, showing the value of combination immunotherapy (57). Similarly, PD-1 blockade can enhance T cell tumor infiltration, increase T cell activation, and improve survival of mice bearing B16 tumors following vaccination with GM-CSF-secreting vaccine (58). There is hope that this combination strategy may be effective for treating aggressive pancreatic cancer as well. A preclinical study by Soares et al. showed that PD-1 blockade in combination with GM-CSF-secreting vaccine prolonged survival of mice bearing Panc02 murine pancreatic ductal adenocarcinomas and this was associated with increased CD8^+^ T cell infiltration into the tumor bed (50). Other studies have employed tumor vaccination with multiple checkpoint inhibitors. Duraiswamy et al. showed that GM-CSF secreting vaccination with PD-1 and CTLA-4 blockade induced rejection of all CT26 tumors and 75% of ID8-VEGF tumors (59). Likewise, Curran et al. showed that GM-CSF or Flt3-ligand secreting vaccines synergized with dual PD-1 and CTLA-4 blockade to prolong survival and increase the ratio of effector to regulatory cells within the murine tumor microenvironment (60).

In patients, two phase I studies using nivolumab in combination with peptide vaccines targeting melanocyte differentiation antigens have shown the relative safety of combining checkpoint blockade with vaccines (61, 62). Taking it a step further, two phase I trials are currently in development testing neoantigen vaccines in combination with blocking antibodies to CTLA-4 or PD-1 in various cancers (NCT02950766; NCT02897765). These initial studies will yield valuable insight into the efficacy of targeting non self-antigens in combination with established immunotherapies.

Looking further ahead, several other T cell costimulatory molecules are also being targeted for immunotherapy. LAG3, TIM3, BTLA, and TIGIT are some of the negative regulators for which inhibitors are currently under investigation (63, 64). Drugs targeting costimulatory molecules like OX-40 and 4-1BB are also under investigation for cancer immunotherapy (65). Rather than “releasing the brake,” drugs targeting this class of molecules would push T cells toward activation (66). This next generation of checkpoint modulatory molecules may further enhance the efficacy of tumor vaccines and provide countless potential combination regimens for testing.

## Combining Vaccines with Therapies Targeting Regulatory Immune Cells

Inhibitory ligands are not the only mechanism suppressing antitumor T cell responses. Solid tumors are often inundated with regulatory immune cells such as Foxp3^+^ T regulatory cells (Treg) and MDSCs.

Tregs can suppress conventional T cell responses by several mechanisms. With their high expression of the IL-2 receptor α chain (CD25), Tregs are thought to act as IL-2 sinks, thereby reducing the availability of IL-2 for conventional T cells (67). In addition, Tregs can secrete anti-inflammatory cytokines such as IL-10 and TGF-β, regulate adenosine metabolism through CD39 and CD73, and delete effector T cells through granzyme-mediated killing (68–70). They also express CTLA-4 which can outcompete receptors on conventional T cells for interactions with antigen presenting cells (69, 70). Recent work has suggested that CTLA-4 expression on Tregs may account for at least part of the antitumor activity of CTLA-4 blocking antibodies, highlighting the therapeutic relevance of this cell population (71–73). Complicating the issue of Tregs in cancer progression, a recent meta-analysis revealed that increased frequencies of Foxp3^+^ Tregs correlated with decreased overall survival in several cancers, but correlated with a better prognosis in head and neck, esophageal, and colorectal cancers (74).

The recruitment of Tregs to the tumor is at least partly directed by CD8^+^ T cells within the tumor, suggesting that vaccine-induced T cells may inadvertently increase intratumoral Treg numbers (47). Direct experimental evidence has confirmed that depletion of Tregs using a genetically engineered Diphtheria toxin receptor mouse can delay melanoma tumor growth, and dramatically improved efficacy of a melanoma-targeted vaccine (75). This illustrates the potential benefit of using a tumor vaccine in combination with therapies targeting Tregs within the tumor microenvironment. In fact, the long appreciated synergy between some chemotherapeutic agents, such as cyclophosphamide and cancer immunotherapies, may be partly attributed to a reduction in Tregs (76). Therapies specifically targeting Tregs are also under development. Denileukin diftitox is an IL-2/Diphtheria toxin fusion product designed to deplete CD25^hi^ Tregs (77). However, a recent study in melanoma patients established that a single dose of denileukin diftitox prior to vaccination with a peptide vaccine did not enhance response to the vaccine (78). Another therapeutic avenue under research is peptide inhibitors of Foxp3 (79). In two preclinical studies, peptide inhibitors of Foxp3 enhanced the antitumor efficacy of a murine tumor vaccine (79, 80). Though early in development, these studies are enticing, given their potential to modulate Tregs specifically. Other strategies to target Tregs, including antibodies against GITR and CCR4, are currently being tested in patients (NCT02946671; NCT01239134) (81, 82).

Myeloid-derived suppressor cells are immature myeloid cells that can be subdivided into two subgroups based on their similarity to granular polymorphic neutrophils (PMN-MDSC) or monocytes (M-MDSC). Both of these exhibit potent yet distinct immunosuppressive effects on T cells through production of immunosuppressive molecules, such as Arginase I and iNOS, and anti-inflammatory cytokines, such as TGF-β and IL-10 (83). The importance of MDSCs in cancer progression was highlighted in a recent meta-analysis showing a significant correlation between MDSC frequency in blood and overall survival in several solid tumors (84). Different treatment modalities can dramatically influence MDSC function. Cyclophosphamide can increase the numbers of MDSCs both in mouse models and in humans, while the anthracycline doxorubicin can reduce the burden of MDSCs in a mouse model of melanoma (85–87). In the context of immunotherapy, Hosoi et al. demonstrated in a mouse model of melanoma that tumors rapidly adapt to T cell attack by recruiting M-MDSCs in an IFNγ-dependent process (88). Prevention of this MDSC recruitment in CCR2^−/−^ mice significantly improved the antitumor T cell response (88). Therapies targeting MDSCs are an active area of research and their combination with tumor vaccines may remove another roadblock for antitumor T cells generated by a vaccine. The role of macrophages in eliminating pathogens is well-established; however, their impact on tumor progression is more controversial (89–92). Tumor-associated macrophages (TAM) are highly prevalent in tumor tissues, and the overlap between these cells and M-MDSCs can be difficult to distinguish (91, 92). Several studies have suggested that TAMs can stifle antitumor T cells. DeNardo et al. showed that TAM frequency correlated with poorer prognosis in patients with breast cancer, and TAM depletion following chemotherapy resulted in expansion of intratumoral T cells in a mouse model (93). Similarly, in a seminal study dissecting the cellular origin of TAMs in a mouse mammary tumor model, Franklin et al. showed that depletion of TAMs increased the frequency of granzyme B^+^ T cells within the tumor, suggesting that TAMs limit T cell cytotoxicity (91). The exact mechanisms behind these findings are likely multifactorial and may overlap with those used by MDSCs (92). Further complicating this topic, TAMs are highly plastic cells and can take on pro-inflammatory (M1) and anti-inflammatory (M2) phenotypes (94). More recent studies have started to investigate methods for promoting immunostimulatory TAM phenotypes. Georgoudaki et al. showed that anti-MARCO antibodies could reprogram TAMs toward an immunostimulatory phenotype through an FcγRIIB-dependent mechanism and thereby limit tumor growth (95). Modulating TAM phenotype may also be an effective adjuvant therapy to enhance the efficacy of vaccine-induced T cells.

## Other Potential Mechanisms of Vaccine Failure

Apart from immunological signaling receptors and regulatory immune cells, other environmental factors within the tumor can dramatically influence tumor-specific T cell function. Hypoxia is a common characteristic of advanced solid tumors with localized variation within individual tumors (96). Limited data exist directly testing the influence of hypoxia on antitumor T cells *in vivo*. However, in one recent preclinical study, Scharping et al. showed that metformin can enhance the efficacy of PD-1 blockade by modulating intratumoral hypoxia and increase the production of effector cytokines by intratumoral lymphocytes (97). This study suggests that tumor hypoxia may be another pathway to modulate in conjugation with tumor vaccines. However, this idea is complicated by the recent observation that excessive oxygenation in some tissues can promote antitumor T cell dysfunction. In a study by Clever et al., mice lacking the oxygen sensing prolyl-hydroxylase proteins in their T cells were protected from pulmonary melanoma metastases, showing that oxygen can suppress antitumor T cell effects in some tissues (98). Overall, tissue oxygenation likely has a complicated role in modulating T cell activity but may represent another therapeutic avenue to be utilized in conjunction with tumor vaccination.

Indoleamine 2,3-dioxygenase 1 (IDO) is a cytosolic IFNγ-regulated enzyme responsible for the local degradation of tryptophan (47, 99–101). Through this mechanism, IDO can limit antitumor immunity by inhibiting effector T cell function and/or promoting Treg activity (102–104). Several IDO inhibitors have been evaluated including 1-methyltryptophan (1MT) (105). IDO inhibition with 1MT has been shown to delay murine tumor growth when combined with a blocking antibody to CTLA-4 (106, 107). In a recent study examining both murine and canine tumors, antitumor effects of “*in situ*” vaccination (consisting of intratumoral CpG and radiotherapy) were greatly improved by 1MT administration, as evidenced by delayed tumor growth and decreased intratumoral Tregs (108). Several clinical trials are testing IDO inhibitors alone or in combination with other immunotherapies (109). Two are currently underway examining IDO inhibitors in combination with vaccination. One is studying a DC vaccine in combination with 1MT in patients with metastatic breast cancer (NCT01042535); another is testing a peptide vaccine in combination with a novel IDO inhibitor in patients with melanoma (NCT01961115). Results from these and future studies may justify the combined immunotherapy of vaccination and IDO inhibition.

A landmark study has recently revealed that tumor necrosis can suppress antitumor T cell activity (110). Following tumor cell necrosis, intracellular potassium ions (K^+^) are released into the extracellular space and accumulate within antitumor T cells. This increased K^+^ limits antitumor T cell function by suppressing Akt activation, thereby allowing tumor immune evasion (110). This novel mechanism of immunosuppression may represent yet another potential roadblock for tumor vaccine success. Methods to limit K^+^-mediated immunosuppression may also synergize with tumor vaccines by allowing full effector function of vaccine-induced T cells within the tumor microenvironment.

Increasing evidence shows that non-immune factors influence the activity of antitumor T cells within a hostile microenvironment. Altering local tissue factors, such as hypoxia, tryptophan, and extracellular ions, may ensure that vaccine-induced T cells retain their metabolic fitness within the tumor itself and thereby increase the clinical efficacy of tumor vaccines.

## Summary

**Figure 1:**
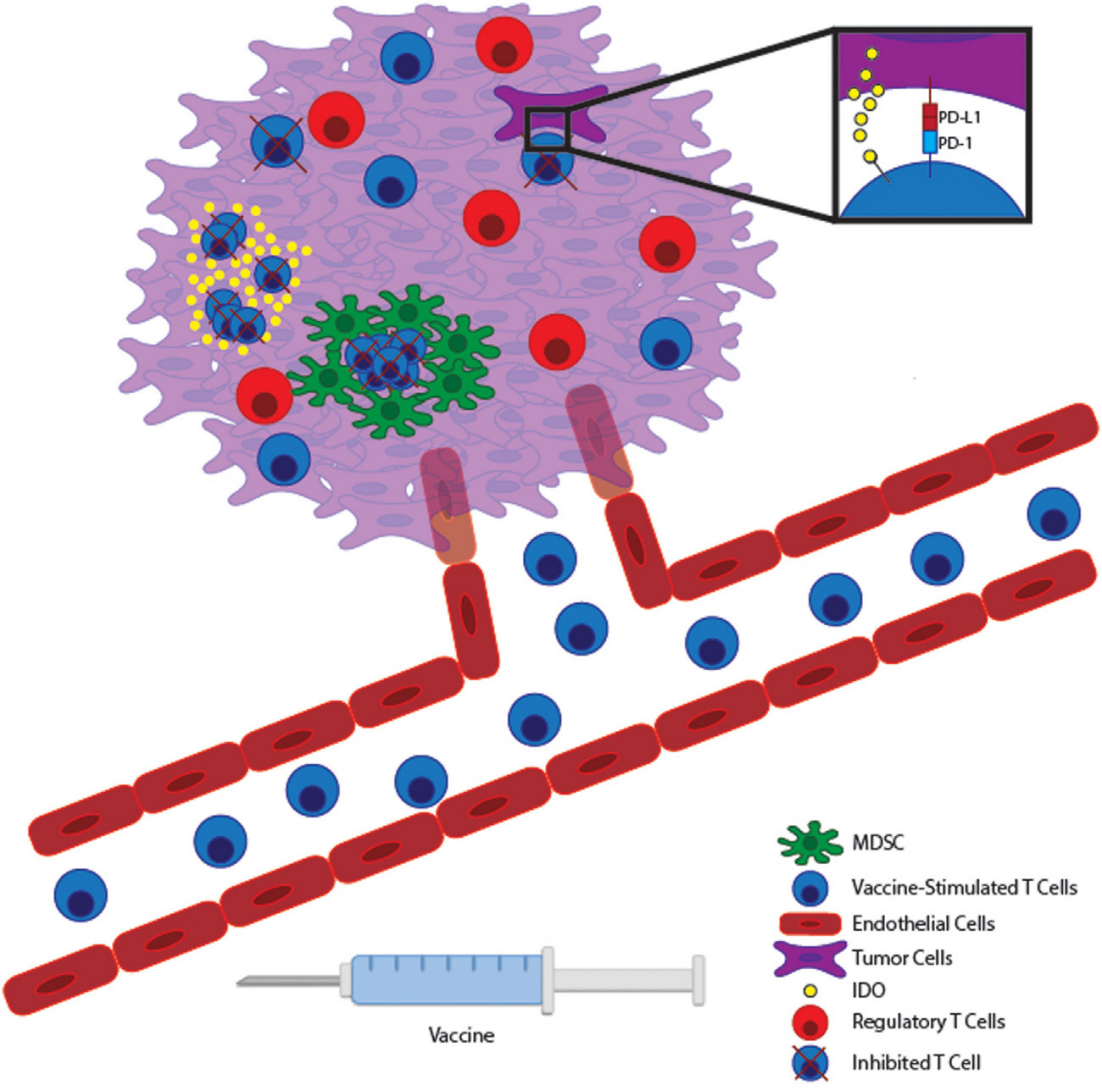
Vaccine-stimulated T cells encounter many immunosuppressive pathways within the tumor microenvironment. Immunogenic tumor vaccines will stimulate tumor-reactive T cells to expand within lymphoid tissue and migrate through the vasculature to the tumor bed. Within malignant tissue, T cells can potentially encounter many immunosuppressive pathways. Tumor cells can express inhibitory molecules, such as PD-L1 and IDO, which suppress T cell effector function and allow tumor immunoevasion. At the same time, many tumors promote the accumulation of regulatory immune cells, such as myeloid-derived suppressor cells (MDSCs) and Tregs to further inhibit effector T cell function locally. Therapies targeting these local immunoevasive pathways may allow vaccine-stimulated T cells to maintain full functionality within a hostile tumor microenvironment.

Decades of research have shown both in preclinical models and in patients that T cells can be trained to target tumor antigens. Unfortunately, the majority of clinical studies of cancer vaccines have shown at best only modest benefit. This may be partly attributed to the choice of antigen and the harsh immunosuppressive nature of the tumor microenvironment (Figure 1). Targeting neoantigens is a highly encouraging prospect that may limit the dangers of autoimmunity while enhancing the magnitude of the antitumor response. However, based on early studies following this approach, it is unlikely that vaccination alone will cure as neoantigen-specific T cells will still be subject to the hostile tumor microenvironment. Combining effective vaccines with therapies aimed at the tumor microenvironment will likely yield optimal results. After all, combination therapy has been the hallmark of the chemotherapeutic revolution as well.

## Author Contributions

JMG and KMK wrote the paper with help from STY.

## Conflict of Interest Statement

The authors declare that the research was conducted in the absence of any commercial or financial relationships that could be construed as a potential conflict of interest.
